# Treatment-related lymphopenia predicts long-term survival in locally advanced head and neck cancer: *Post-Hoc* analysis of a randomized phase III trial

**DOI:** 10.3389/fimmu.2026.1805437

**Published:** 2026-08-31

**Authors:** Gulidanna Shayan, Xin Guo, Xiaodong Huang, Kai Wang, Yuan Qu, Ye Zhang, Runye Wu, Xuesong Chen, Jingwei Luo, Jianghu Zhang, Jingbo Wang, Junlin Yi

**Affiliations:** 1 Department of Radiation Oncology, National Cancer Center/National Clinical Research Center for Cancer/Cancer Hospital, Chinese Academy of Medical Sciences and Peking Union Medical College, Beijing, China; 2 Department of Radiation Oncology, National Cancer Center/National Clinical Research Center for Cancer/Hebei Cancer Hospital, Chinese Academy of Medical Sciences (CAMS), Langfang, Hebei, China

**Keywords:** absolute lymphocyte count, head and neck squamous cell carcinoma, nomogram, radiation therapy, randomized clinical trial

## Abstract

**Purpose:**

To evaluate the prognostic value of treatment related decline of peripheral absolute lymphocyte count (ALC) in patients with locally advanced head and neck squamous cell carcinoma (LA-HNSCC) after preoperative radiation therapy (RT) with or without chemotherapy.

**Methods:**

This *post-hoc* analysis was performed using data of 222 patients from a phase III, randomized controlled trial in LA-HNSCC. ALCs were measured before, during, and after RT. The ALC decline was defined as the difference between pre-treatment ALC and the nadir ALC observed during RT. Optimal cut-off values for hematologic and nutritional indices were determined by ROC analysis. Survival outcomes were estimated using Kaplan-Meier analysis and compared with the log-rank test. Prognostic factors were identified using LASSO regression and multivariable Cox models. A nomogram was constructed based on independent prognostic variables.

**Results:**

With a median follow-up of 132.0 (IQR: 104.17-151.73) months, no significant difference was observed in the 10-year overall survival (OS) and progression-free survival (PFS) between treatment groups. Lymphopenia during RT occurred in 197 patients (88.7%), including grade 3 in 112 (50.5%) and grade 4 in 10 (4.5%). An ALC decline > 0.74×10^9^/L was independently associated with poorer OS and PFS. The nomogram integrating T stage, N stage, ALC decline, and neutrophil-to-albumin ratio (NAR) yielded a C-index of 0.66, a 5-year AUC of 0.738, good calibration, and favorable net benefit on DCA.

**Conclusion:**

Treatment-related ALC decline is an independent adverse prognostic factor in LA-HNSCC. A nomogram incorporating ALC decline performs favorably in individualized survival prediction.

## Introduction

Head and neck squamous cell carcinoma (HNSCC), encompassing malignancies of the oral cavity, oropharynx, hypopharynx, and larynx, remains a therapeutic challenge despite advances in multimodality treatment (1–3). Current standards of care for locally advanced HNSCC (LA-HNSCC) include chemoradiotherapy or surgery followed by risk-adapted radiotherapy with or without chemotherapy. However, long-term outcomes remain dismal, with 5-year survival rates persistently below 40% (2). A prior randomized phase 3 trial showed that preoperative concurrent chemoradiotherapy with cisplatin significantly improved distant metastasis-free survival compared to preoperative radiotherapy alone (4). While this highlights the potential of systemic therapy integration, the persistent mortality gap underscores the need for novel prognostic biomarkers to refine therapeutic strategies.

With recent advances of immunotherapy in HNSCC, studies suggested that immune status, particularly the count and function of lymphocytes, played a crucial role in determining cancer prognosis and treatment efficacy (5, 6). In several solid tumors, such as lung, esophageal, and pancreatic cancers, treatment-induced lymphopenia has been associated with poorer survival outcomes (7, 8). Lymphocytes are known to be extremely radiosensitive with an LD50 (lethal dose required to inactivate 50% of cells) as low as 2 Gy (9). Treatment-related absolute lymphocyte count (ALC) decline, defined as the reduction from baseline to the nadir during radiotherapy, may therefore have prognostic implications. Unlike the absolute nadir ALC, which represents a static lowest value during treatment and may be influenced by pretreatment lymphocyte levels, ALC decline captures the within-patient dynamic reduction from baseline to the lowest on-treatment value. Therefore, ALC decline may more directly reflect the magnitude of treatment-related immune depletion and provide individualized prognostic information beyond nadir ALC alone. However, its prognostic value in patients with HNSCC receiving RT remains inadequately characterized.

Leveraging patient-level data from a randomized phase III trial with long-term follow-up, this *post-hoc* study aimed to determine whether ALC decline could serve as an independent biomarker for long-term survival and to explore its potential role in improving risk stratification for patients with LA-HNSCC. We further evaluated a panel of immune and nutritional indices to compare their prognostic relevance with that of ALC decline. This investigation could assist risk stratification in LA-HNSCC and inform personalized approaches to improve survival in this challenging patient population.

## Methods

### Study population

This study was approved by the institutional review board of our hospital and informed consent was obtained from patients. This was a *post-hoc* analysis of the data from a phase III, randomized trial which was conducted between September 2002 to May 2012. A total of 222 HNSCC patients were enrolled, among whom 104 patients were assigned to the preoperative concurrent chemoradiotherapy group (pre-S CRT) and 118 patients were assigned to the preoperative radiotherapy group (pre-S RT). The details of the study design and data collection have been published in the previous study (4). Peripheral blood samples were prospectively collected at multiple time points, including prior to radiotherapy (pre-RT ALC), weekly during RT, and at scheduled intervals after RT (1, 3, and 6 months, and 1 year post-RT). All enrolled patients underwent routine complete blood count and serum biochemical testing before the initiation of RT and on a weekly basis throughout the treatment course. For the current analysis, patients with available peripheral blood-based ALCs prior to RT and during or after RT were included. All 222 patients from the original trial with available pretreatment and on-treatment ALC data were included in the present analysis. No patients were excluded due to missing ALC data for the hematologic parameters used in this study. The original randomized phase III trial was registered under Clinical trial information: ChiCTR-TRC-114004322.

### Treatment

The initial radiotherapy regimen was identical in both treatment arms: 50 Gy in 25 daily fractions was prescribed to the primary tumor and nodal disease. Radiotherapy was delivered using either conventional radiotherapy or IMRT with 6-MV photons. In the preS-CRT group, cisplatin 30 mg/m² once weekly was administered starting on the first day of radiotherapy. Tumor response was assessed at the end of the fifth week, after completion of 50 Gy. Responders, defined as patients with ≥80% reduction of the primary lesion, received a boost to the primary gross tumor volume to a total dose of 70 Gy in 35 daily fractions, with concurrent weekly cisplatin in the preS-CRT group. Non-responders underwent resection of the primary tumor and modified neck dissection 4–6 weeks after completion of preoperative 50 Gy (4).

### Definitions and outcomes

Lymphopenia was graded using the Common Terminology Criteria for Adverse Events version 5.0 (CTCAE 5.0). The lowest ALC during RT was defined as the nadir ALC. ALC decline was defined as the difference between pre-treatment ALC and the nadir ALC observed during RT. Relative ALC decline was calculated as (baseline ALC - nadir ALC)/baseline ALC to account for baseline lymphocyte levels. The optimal cutoff for absolute and relative ALC decline was determined using ROC analysis. The prognostic performance of absolute and relative ALC decline based models was compared using the C-index and time-dependent AUCs at 1, 3, and 5 years. The timing of nadir ALC was further evaluated using the interval from the start of radiotherapy to the date of nadir ALC, the interval from nadir ALC to the end of radiotherapy, and the relative timing of nadir ALC within the entire radiotherapy course. Early post-treatment lymphocyte recovery was assessed using ALC values measured approximately one month after completion of radiotherapy. Lymphocyte recovery was defined as one-month post-treatment ALC ≥0.8 × 10^9^/L, according to the laboratory lower limit of normal.

A panel of immune and nutritional indices, such as neutrophil-to-lymphocyte ratio (NLR), neutrophil-to-albumin ratio (NAR), platelet-to-lymphocyte ratio (PLR), systemic inflammation score (SIS), prognostic nutritional index (PNI), and geriatric nutritional risk index (GNRI) were also collected and assessed for their prognostic relevance with multiple survival indices. The full list of definitions and formulas for the immune and nutritional indices are provided in **Supplementary Table 1**.

Progression free survival (PFS), distant metastasis free survival (DMFS), local recurrence free survival (LRFS), and overall survival (OS) were evaluated and calculated from the date of randomization. PFS events included locoregional recurrence, distant metastasis, or death, whichever occurred first. DMFS events included distant metastasis or death, LRFS events included local recurrence or death, and OS events included death from any cause.

### Statistical analysis

Continuous variables were compared between groups using the Mann-Whitney U test. Categorical variables were compared using chi-square tests. Age, ALCs, NLR, NAR, PLR, SIS, BMI, PNI, and GNRI were investigated as categorical variable. The optimal cut-offs were determined based on 3-year OS using the optimal receiver operating characteristics (ROC) in R software. Restricted cubic spline (RCS) regression with 4 knots was applied to evaluate the potential non-linear association between the variables and OS and PFS, with non-linearity assessed by Wald test and corresponding p values reported. Survival probabilities were estimated using the Kaplan-Meier method and compared between groups using the log-rank test. Cox proportional hazards regression models were applied to assess associations between prognostic factors and survival outcomes. Variables with a p-value < 0.15 in univariate analysis, along with clinically relevant covariates (gender, age, T stage, and N stage), were enrolled into the multivariable model. Hazard ratios (HRs) and 95% confidence intervals (CIs) were calculated. The proportional hazards assumption was assessed using Schoenfeld residual tests and residual plots for the multivariable Cox models. Global Schoenfeld test p values were reported for each model.

To develop the prognostic model, least absolute shrinkage and selection operator (LASSO) regression with 10-fold cross-validation was applied to the candidate variables to select predictive features with most significance and to minimize the model overfitting. The selected predictors were incorporated into a multivariable Cox proportional hazards model to construct a nomogram for estimating 3-year PFS.

Internal validation of the nomogram was performed using bootstrap resampling (B = 500). Model discrimination was quantified by the concordance index (C-index). Calibration was assessed by plotting predicted versus observed survival probabilities after bootstrap correction. Clinical utility was evaluated by decision curve analysis (DCA) across a range of threshold probabilities. Time-dependent ROC curves and the corresponding area under the curve (AUC) were also generated to assess predictive accuracy over time.

A two-sided p-value < 0.05 was considered statistically significant. All statistical analyses were performed using SPSS version 22.0 (IBM Corp., Armonk, NY, USA) and R version 4.1.2 (https://www.r-project.org/). The following R packages were used: survival, survminer, rms, glmnet, dplyr, readr, ggplot2, timeROC, and rmda.

## Results

### Patients baseline characteristics

A total of 222 patients were analyzed, including 118 in the preS-RT group and 104 in the preS-CRT group. Baseline characteristics were generally comparable (**Table 1**), except for a higher proportion of patients with T4a disease (49.0% vs. 30.5%, p = 0.045). Detailed description of each characteristics has been reported in the previous publication (4).

**Table 1:** Patient baseline characteristic.

Characteristic	Level	Total (n=222)	preS-RT (n=118)	preS-CRT (n=104)	p-value
age (%)	<55	143 (64.4%)	69 (58.5)	74 (71.2)	0.067
	>=55	79 (35.6%)	49 (41.5)	30 (28.8)	
sex (%)	male	190 (85.6%)	105 (89.0)	85 (81.7)	0.179
	female	32 (14.4%)	13 (11.0)	19 (18.3)	
Primary site	Oral cavity	28(12.6%)	14 (11.9)	14 (13.5)	0.52
	Oropharynx	66(29.7%)	36 (30.5)	30 (28.8)	
	Hypopharynx/Larynx	128(57.7%)	68 (57.6)	60 (57.7)	
T (%)	1	12 (5.4%)	6 (5.1)	6 (5.8)	0.045
	2	43 (19.4%)	25 (21.2)	18 (17.3)	
	3	65 (29.3%)	43 (36.4)	22 (21.2)	
	4a	87 (39.2%)	36 (30.5)	51 (49.0)	
	4b	15 (6.8%)	8 (6.8)	7 (6.7)	
N (%)	0	28 (12.6%)	19 (16.1)	9 (8.7)	0.357
	1	42 (18.9%)	22 (18.6)	20 (19.2)	
	2a	9 (4.1%)	4 (3.4)	5 (4.8)	
	2b	63 (28.4%)	35 (29.7)	28 (26.9)	
	2c	50 (22.5%)	21 (17.8)	29 (27.9)	
	3	30 (13.5%)	17 (14.4)	13 (12.5)	
stage (%)	III	35 (15.8%)	21 (17.8)	14 (13.5)	0.623
	IVa	143 (64.4%)	73 (61.9)	70 (67.3)	
	IVb	44 (19.8%)	24 (20.3)	20 (19.2)	
RTtech (%)	3D-CRT	124 (55.9%)	62 (52.5)	62 (59.6)	0.356
	IMRT	98 (44.1%)	56 (47.5)	42 (40.4)	
SIS (%)	low (≤ 715.2)	123 (55.4%)	64 (54.2)	59 (56.7)	0.812
	high (> 715.2)	99 (44.6%)	54 (45.8)	45 (43.3)	
nadirALC (%)	low (≤ 0.73 × 10^9^/L)	191 (86.0%)	93 (78.8)	98 (94.2)	0.002
	high (> 0.73 × 10^9^/L)	31 (14.0%)	25 (21.2)	6 (5.8)	
ALCdecline (%)	low (≤ 0.74 × 10^9^/L)	50 (22.5%)	31 (26.3)	19 (18.3)	0.207
	high (> 0.74 × 10^9^/L)	172 (77.5%)	87 (73.7)	85 (81.7)	
NLR (%)	low (≤ 2.01)	56 (25.2%)	32 (27.1)	24 (23.1)	0.591
	high (> 2.01)	166 (74.8%)	86 (72.9)	80 (76.9)	
PNI (%)	low (≤ 60)	198 (89.2%)	105 (89.0)	93 (89.4)	1
	High (>60)	24 (10.8%)	13 (11.0)	11 (10.6)	
BMI (%)	low (≤ 23.59)	127 (57.2%)	63 (53.4)	64 (61.5)	0.276
	high(> 23.59)	95 (42.8%)	55 (46.6)	40 (38.5)	
PLR (%)	low (≤ 148.57)	126 (56.8%)	64 (54.2)	62 (59.6)	0.502
	high(> 148.57)	96 (43.2%)	54 (45.8)	42 (40.4)	
NISI (%)	low (≤ 0.75)	219 (98.6%)	116 (98.3)	103 (99.0)	1
	high(> 0.75)	3 (1.4%)	2 (1.7)	1 (1.0)	
PAR (%)	low (≤ 6.2)	157 (70.7%)	82 (69.5)	75 (72.1)	0.779
	high (> 6.2)	65 (29.3%)	36 (30.5)	29 (27.9)	
SAR (%)	low(≤ 7.74)	216 (97.3%)	115 (97.5)	101 (97.1)	1
	high (> 7.74)	6 (2.7%)	3 (2.5)	3 (2.9)	
WAPNI (%)	low(≤ 2.81)	200 (90.1%)	108 (91.5)	92 (88.5)	0.591
	high (> 2.81)	22 (9.9%)	10 (8.5)	12 (11.5)	
CIMI (%)	low(≤ 6.19)	177 (79.7%)	101 (85.6)	76 (73.1)	0.032
	high (> 6.19)	45 (20.3%)	17 (14.4)	28 (26.9)	
AHR (%)	low (≤ 0.33)	173 (77.9%)	90 (76.3)	83 (79.8)	0.637
	high (> 0.33)	49 (22.1%)	28 (23.7)	21 (20.2)	
GNRI (%)	low (≤ 105.91)	112 (50.5%)	56 (47.5)	56 (53.8)	0.415
	high (> 105.91)	110 (49.5%)	62 (52.5)	48 (46.2)	
NAR (%)	low (≤ 1)	94 (42.3%)	51 (43.2)	43 (41.3)	0.884
	high (> 1)	128 (57.7%)	67 (56.8)	61 (58.7)	

SIS, Systemic Inflammation Score; Nadir ALC, lowest Absolute Lymphocyte Count during treatment; ALC decline, Absolute Lymphocyte Count decline; NLR, Neutrophil-to-Lymphocyte Ratio; PNI, Prognostic Nutritional Index; NRI, Nutritional Risk Index; BMI, Body Mass Index; PLR, Platelet-to-Lymphocyte Ratio; NISI, Nutritional-Inflammatory Status Index; PAR, Platelet-to-Albumin Ratio; SAR, Serum Iron-to-Albumin Ratio; WAPNI, Weight-Adjusted Prognostic Nutritional Index; CIMI, Combined Iron Metabolism Index; AHR, Albumin-to-Hemoglobin Ratio; GNRI, Geriatric Nutritional Risk Index; NAR, Neutrophil-to-Albumin Ratio.

### Immune and nutritional indices

The distribution of general characteristics and immune-nutritional indices is presented in **Table 1**. Among the 222 patients, 197 (88.7%) developed lymphopenia during radiation therapy. Notably, 112 patients (50.5%) developed grade 3 lymphopenia, and 10 (4.5%) developed grade 4 lymphopenia. Cut-off values for all indices were derived from ROC analyses, while RCS confirmed no evidence of non-linear associations for SIS, NAR, and ALC decline (**Supplementary Figure 1**). Optimal ROC analysis identified ALC decline of 0.74×10^9^/L as the optimal threshold. Most indices were comparable across treatment groups, with the exception of a significantly lower nadir ALC (94.2% vs. 78.8%, p = 0.002) and a higher proportion of elevated Combined Iron Metabolism Index (CIMI) (26.9% vs. 14.4%, p = 0.032) in the preS-CRT group.

### Ten-year survival outcomes

**Figure 1:**
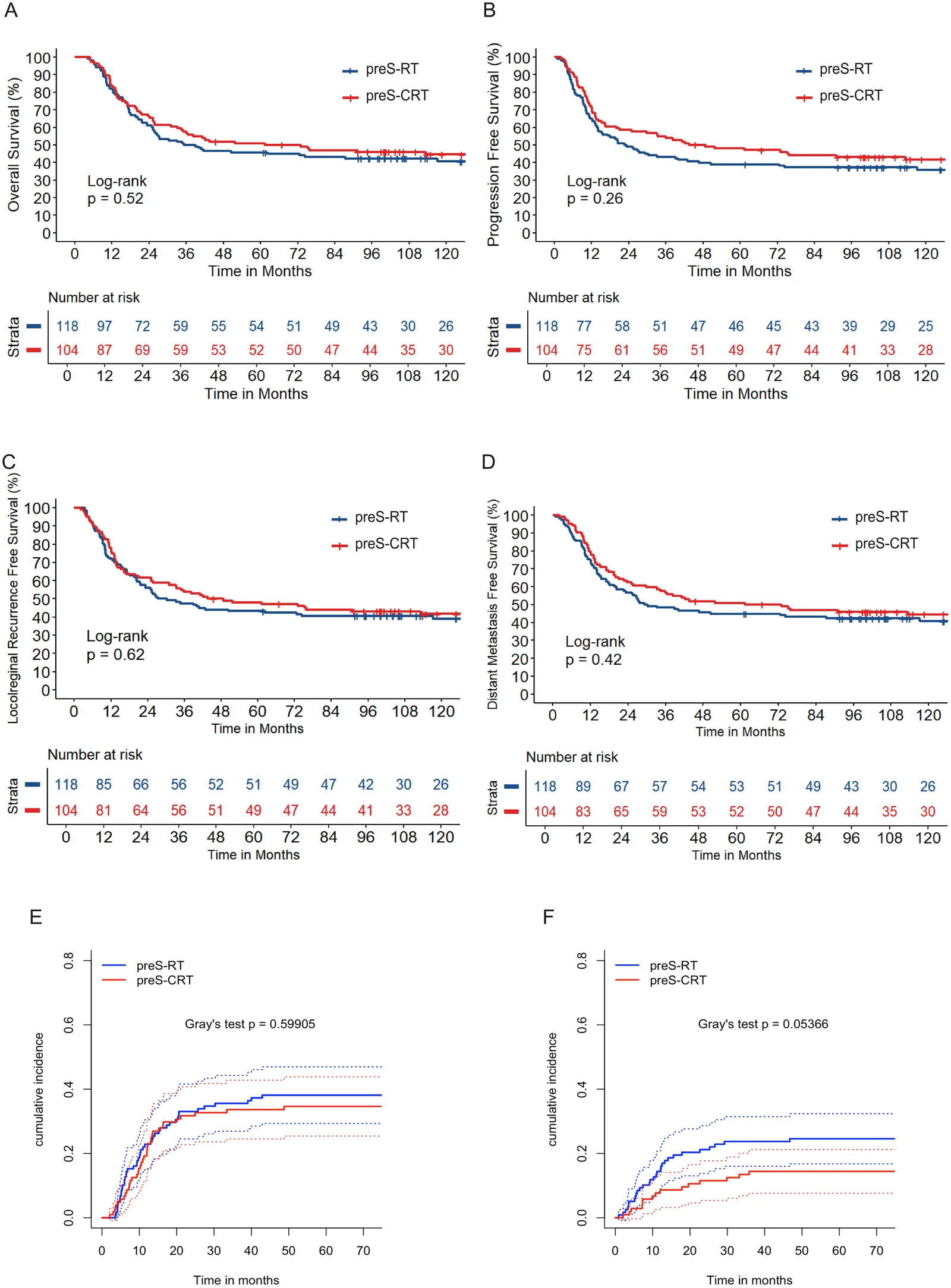
Kaplan–Meier survival and competing risk analyses in patients treated with preS-RT or preS-CRT. **(A)** Overall survival (OS). **(B)** Progression-free survival (PFS). **(C)** Locoregional relapse-free survival (LRFS). **(D)** Distant metastasis-free survival (DMFS). **(E)** Cumulative incidence of locoregional recurrence (LR) with competing risk analysis. **(F)** Cumulative incidence of distant metastasis (DM) with competing risk analysis.

With a median follow-up duration of 132.0 (IQR: 104.17-151.73) months, the 10-year estimate and 95% CI of OS, PFS, LRFS, and DMFS of entire cohort was 42.5%(36.4%-49.7%), 38.6%(32.6%-45.7%), 40.4%(34.3%-47.5%) and 42.5%(36.4%-49.7%), with none survival metrics being significant different between groups (all p > 0.05) (**Figure 1**). Competing risk analysis showed no difference in locoregional recurrence (p = 0.599) and a non-significant trend toward lower distant metastasis in the preS-CRT group (p = 0.054).

### Univariate and multivariable analysis

**Figure 2:**
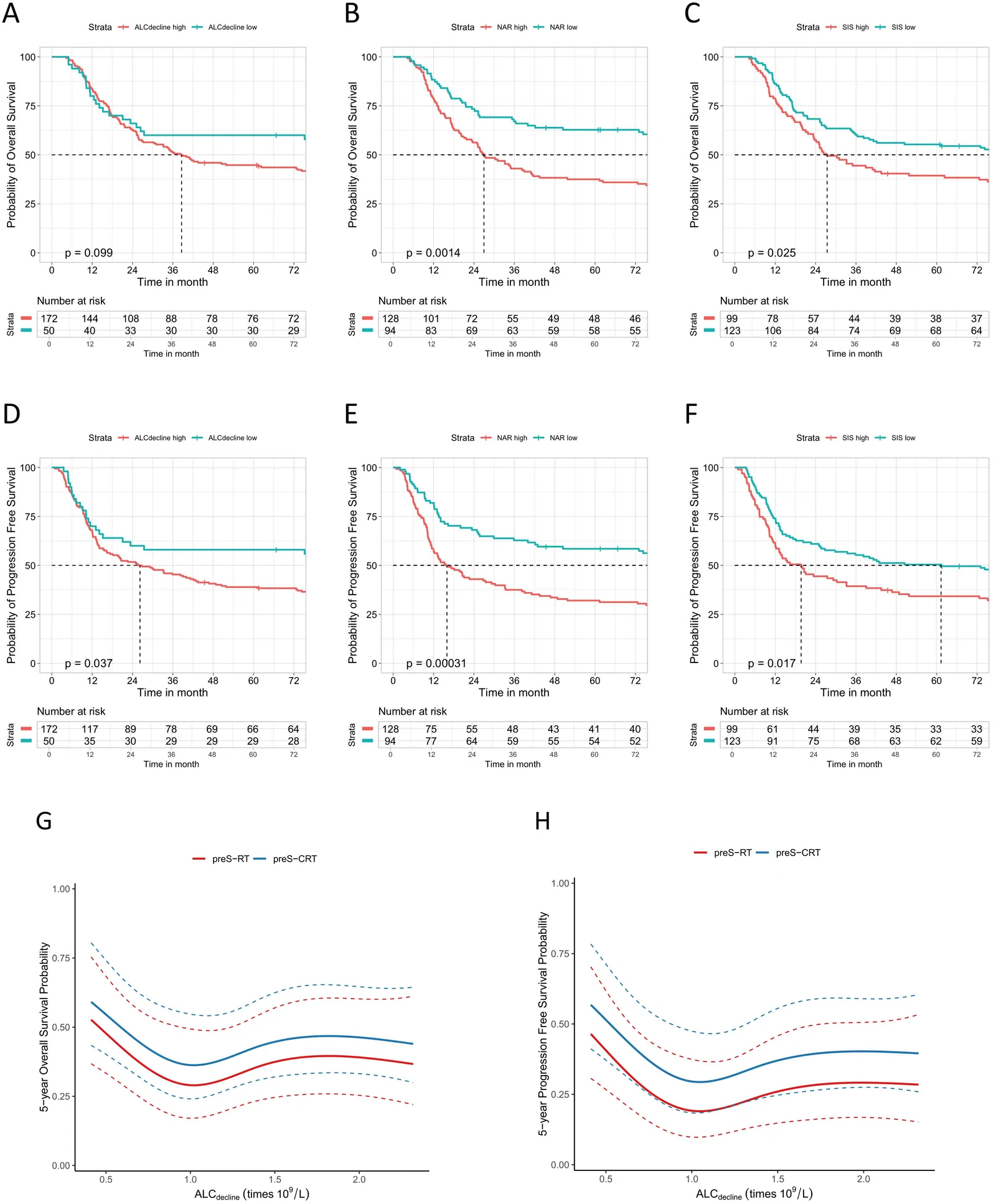
Survival analyses according to immune-nutritional indices. **(A)** OS according to ALC decline (high vs. low). **(B)** OS according to NAR (high vs. low). **(C)** OS according to SIS (high vs. low). **(D)** PFS according to ALC decline (high vs. low). **(E)** PFS according to NAR (high vs. low). **(F)** PFS according to SIS (high vs. low). **(G)** 5-year OS probability according to pretreatment ALC decline, modeled as a continuous variable, with other covariates fixed at T4N2. **(H)** 5-year PFS probability according to pretreatment ALC decline, modeled as a continuous variable, with other covariates fixed at T4N2.

In univariate analysis, an ALC decline ≤ 0.74×10^9^/L was associated with significantly better PFS (HR = 0.62, 95% CI: 0.40-0.98, p = 0.037) and a trend toward improved OS (HR = 0.68, 95% CI: 0.43-1.08, p = 0.099). Higher SIS and higher NAR were linked to poorer OS and PFS (**Figure 2A–F**).

In multivariable analysis, ALC decline ≤ 0.74×10^9^/L remained an independent predictor of favorable OS (HR = 0.55, 95% CI: 0.33-0.91, p = 0.02) and PFS (HR = 0.51, 95% CI: 0.31-0.83, p = 0.01), so did the T stage with regard to OS (HR = 0.56, 95% CI: 0.35-0.91, p = 0.02) and PFS (HR = 0.55, 95% CI: 0.34-0.88, p = 0.01). However, the associations of SIS and NAR with survival outcomes were no longer statistically significant after multivariable adjustment. Details of univariate and multivariable analysis are demonstrated in **Table 2**. No significant global violation was detected by Schoenfeld residual tests and the Schoenfeld residual plots also did not show substantial systematic departures from the proportional hazards assumption (**Supplementary Table 2**).

**Table 2:** Univariate and multivariate analysis for OS and PFS.

Variable	OS				PFS			
	Univariate		Multivariate		Univariate		Multivariate	
	HR(95%CI)	p-value	HR(95%CI)	p-value	HR(95%CI)	p-value	HR(95%CI)	p-value
Group								
preS-RT								
preS-CRT	0.89(0.63-1.26)	0.52			0.82(0.59-1.15)	0.26		
Age								
>55								
<=55	0.83(0.58-1.17)	0.28	0.8(0.56-1.13)	0.21	0.92(0.65-1.28)	0.61	0.89(0.64-1.25)	0.51
Sex								
male								
female	0.89(0.54-1.49)	0.67			0.77(0.46-1.28)	0.31		
T								
T1-2	0.53(0.33-0.83)	0.01	0.56(0.35-0.91)	0.02	0.49(0.31-0.77)	0.00	0.55(0.34-0.88)	0.01
T3-4								
N								
N1-2	0.75(0.52-1.09)	0.13	0.77(0.50-1.21)	0.26	0.76(0.53-1.09)	0.14	0.82(0.54-1.25)	0.37
N3								
stage								
III	0.61(0.36-1.05)	0.08	0.85(0.45-1.64)	0.64	0.54(0.32-0.93)	0.02	0.71(0.38-1.35)	0.30
IVa/b								
RT								
IMRT	0.8(0.56-1.14)	0.21			0.85(0.6-1.2)	0.35		
VMAT								
SIS								
high								
low	0.67(0.48-0.95)	0.03	1.02(0.60-1.73)	0.95	0.67(0.48-0.93)	0.02	1.04(0.63-1.74)	0.87
nadirALC								
high								
low	0.75(0.47-1.21)	0.24			0.87(0.54-1.38)	0.55		
ALCdecline								
high								
low	0.68(0.43-1.08)	0.10	0.55(0.33-0.91)	0.02	0.62(0.4-0.98)	0.04	0.51(0.31-0.83)	0.01
NLR								
high								
low	0.71(0.47-1.08)	0.11	0.82(0.49-1.37)	0.46	0.68(0.46-1.02)	0.06	0.78(0.48-1.27)	0.31
PNI								
high								
low	0.91(0.52-1.59)	0.75			0.91(0.53-1.55)	0.72		
NRI								
high								
low	1.03(0.73-1.46)	0.86			1.02(0.73-1.44)	0.89		
BMI								
high								
low	0.97(0.68-1.37)	0.85			0.85(0.61-1.19)	0.35		
PLR								
high								
low	0.76(0.53-1.07)	0.11	0.74(0.46-1.20)	0.23	0.76(0.55-1.07)	0.11	0.74(0.46-1.17)	0.20
NISI								
high								
low	0.68(0.17-2.75)	0.59			0.84(0.21-3.4)	0.81		
SAR								
high								
low	0.8(0.3-2.16)	0.66			0.89(0.33-2.41)	0.82		
WAPNI								
high								
low	0.84(0.48-1.46)	0.53			0.78(0.46-1.32)	0.36		
CIMI								
high								
low	0.84(0.55-1.27)	0.41			0.8(0.54-1.2)	0.29		
AHR								
high								
low	0.84(0.56-1.26)	0.39			0.81(0.54-1.2)	0.29		
GNRI								
high								
low	1.1(0.78-1.56)	0.58			1.17(0.84-1.64)	0.35		
NAR								
high								
low	0.56(0.39-0.8)	<0.001	0.70(0.44-1.12)	0.14	0.53(0.37-0.75)	<0.001	0.66(0.43-1.04)	0.07

### Interaction and modeling analysis

When ALC decline was analyzed as a continuous variable, no significant interaction was observed between treatment group and ALC decline for either PFS or OS (PFS: p = 0.744; OS: p = 0.517) (**Supplementary Figures 2A, B**; **Supplementary Table 3**). Consistently, categorized interaction analyses using Cox models adjusted for age, sex, T stage, and N stage also showed no significant interaction between treatment group and ALC decline group for OS (interaction HR = 0.67, 95% CI: 0.25-1.75, p = 0.410) or PFS (interaction HR = 0.74, 95% CI: 0.29-1.91, p = 0.535) (**Supplementary Table 4**). Subgroup forest plots further illustrated broadly similar associations between ALC decline and survival outcomes in the preS-RT and preS-CRT groups (**Supplementary Figure 3**). Predicted 5-year OS and PFS probabilities, derived from a multivariable Cox model incorporating T stage, N stage, and ALC decline (fixed at T4N2), showed an overall pattern of decreasing survival probability with increasing ALC decline in both groups (**Figures 2G, H**).

**Figure 3:**
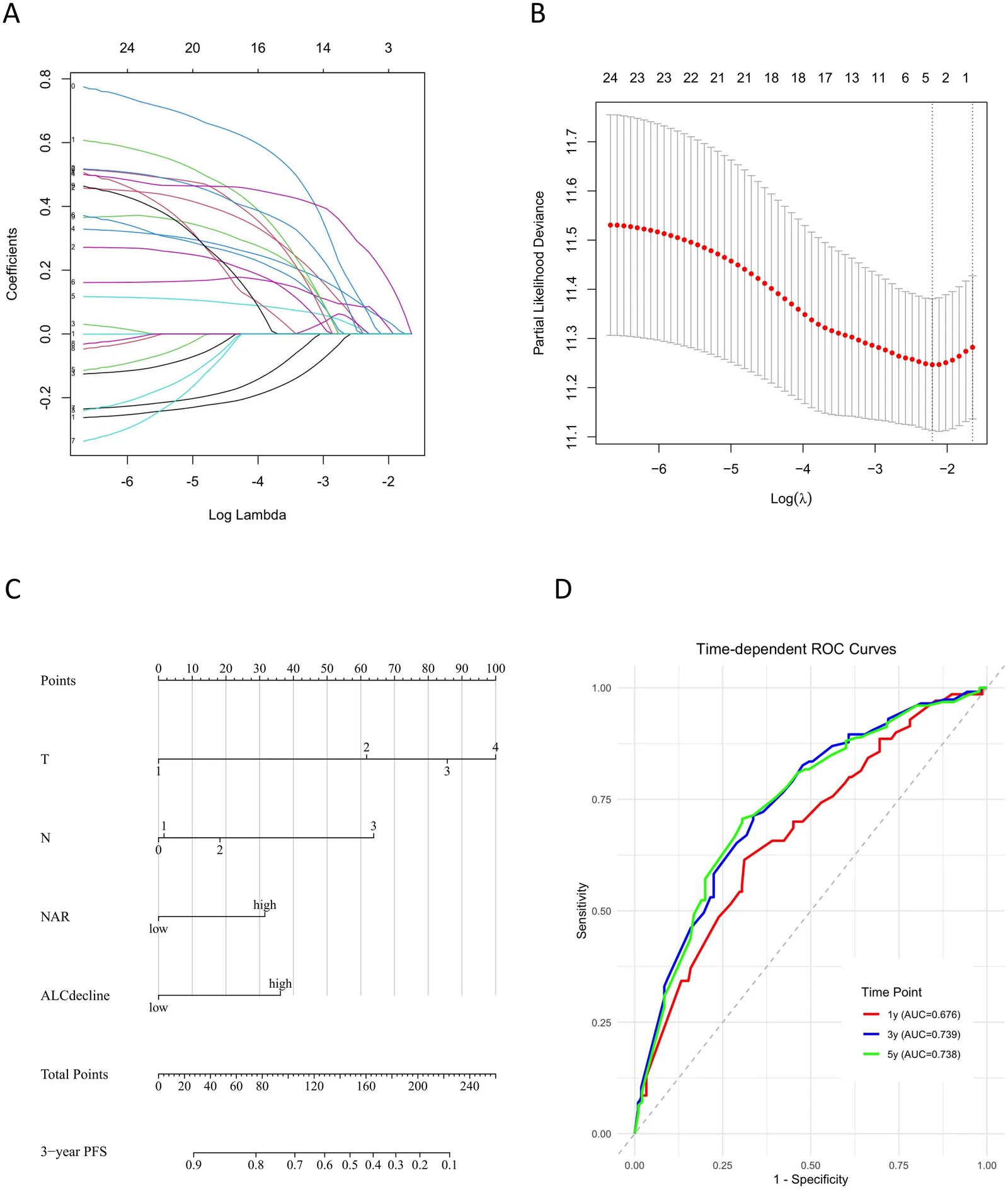
Construction of the prognostic model. **(A)** LASSO coefficient profile of all candidate clinical, immune, and nutritional variables. **(B)** Cross-validation plot for tuning parameter (λ) selection in the LASSO model. **(C)** Nomogram incorporating four selected predictors for estimating 3-year PFS. **(D)** Time-dependent ROC curve assessing the discrimination of the nomogram for 3-year PFS.

**Figure 4:**
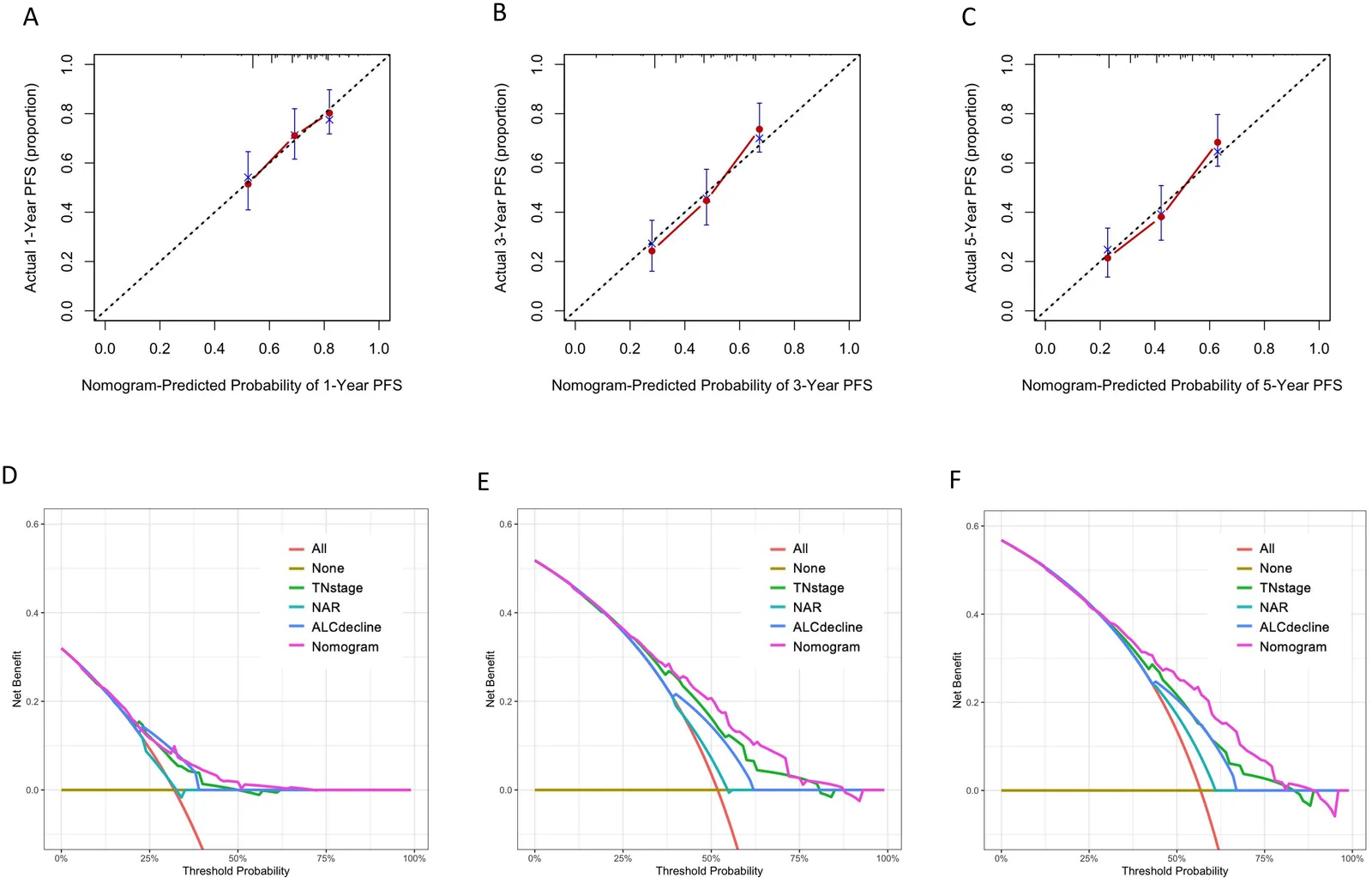
Validation of the prognostic nomogram. **(A–C)** Calibration plots for predicting 1-, 3-, and 5-year PFS. **(D–F)** Decision curve analysis (DCA) of the nomogram at 1, 3, and 5 years.

Using LASSO regression for variable selection, T stage, N stage, NAR, and ALC decline were retained as independent prognostic variables (**Figures 3A, B**). These factors were incorporated into a Cox proportional hazards-based nomogram for individualized prediction of PFS (**Figure 3C**). The model yielded a concordance index (C-index) of 0.66, reflecting moderate discriminatory capacity. Time-dependent ROC analysis demonstrated area under the curve (AUC) values of 0.676, 0.739, and 0.738 for 1-, 3-, and 5-year PFS, respectively (**Figure 3D**). The time-dependent AUC curve is provided in **Supplementary Figure 4**. Calibration plots (**Figures 4A–C**) indicated good agreement between predicted and observed survival probabilities. Decision curve analysis (**Figures 4D–F**) further confirmed the clinical utility of the nomogram, particularly for long-term risk stratification.

Relative ALC decline was additionally calculated as (baseline ALC - nadir ALC)/baseline ALC. The optimal cutoff was 0.887, corresponding to an 88.7% decline. In otherwise identical Cox models incorporating T stage, N stage, NAR, and either absolute or relative ALC decline, the corresponding relative ALC decline model showed a C-index of 0.654 and AUC values of 0.660, 0.726, and 0.716, respectively as shown in **Supplementary Table 5** and **Supplementary Figure 5**.

### Exploratory analysis of ALC decline and predisposing factors

To investigate whether occurrence timing of ALC nadir, radiation parameters and corticosteroid use make an individual more susceptible to developing ALC decline, we conducted additional analyses. Nadir ALC occurred at a median of 32 days after the start of radiotherapy, corresponding to approximately 76% of the radiotherapy course. The median interval from nadir ALC to the end of radiotherapy was 10 days. Overall, 139 of 222 patients (62.6%) reached nadir ALC within the final 2 weeks of radiotherapy, and 87 patients (39.2%) reached nadir ALC within the final week (**Supplementary Table 6**, **Supplementary Figure 6**). Among 172 patients with available one-month post-treatment ALC data, 87 patients (50.6%) recovered to normal level, whereas did not significantly associated with improved OS, PFS, LRFS, or DMFS (**Supplementary Table 7**).

We further explored whether greater ALC decline was associated with larger treatment volumes or available plan-based dosimetric parameters. As shown in **Supplementary Table 8**, no significant differences were observed between the low and high ALC decline groups in GTVp volume, GTVnd volume, total GTV volume, total CTV volume, total PTV volume, or the corresponding mean dose parameters.

Corticosteroid use was recorded in 37 of 222 patients (16.7%) and was generally short-term (range: 3–18 days) during radiotherapy. The proportion of corticosteroid use did not differ significantly between the preS-RT and preS-CRT groups (15/118 [12.7%] vs. 22/104 [21.2%], Fisher’s exact test P = 0.106). Corticosteroid use was not significantly associated with nadir ALC or any survival outcomes, including OS, PFS, LRFS, and DMFS (**Supplementary Table 9**).

## Discussion

In this *post-hoc* analysis of a randomized phase III trial in patients with LA-HNSCC (4), we reported the 10-year survival and unveiled a nearly steady pattern after 5-year follow-up with regard to all survival endpoints. Most notably, as a readily measurable immune parameter, ALC decline during RT was independently associated with both PFS and OS, reinforcing the growing evidence that host immune status, particularly treatment-induced lymphopenia, plays a pivotal role in shaping long-term oncologic outcomes (7, 10).

To our knowledge, this is the first study reporting 10-year survival results in HNSCC, with notably long follow-up (median: 132 months) providing robust long-term outcome data. In the recently updated combined analysis of RTOG 9501 and EORTC 22931, the 7-year OS of postoperative CRT and postoperative RT groups was 41% and 33%, respectively (11). With similar composition of patient cohort, our study achieved a 10-year OS of 42.5%, without significant difference between two preoperative modalities. Besides the advanced RT technique (3DCRT and IMRT) in our study, the sequence of preoperative implementation of RT or CRT may also contribute to the more favorable long-term outcome, due to the potential advantages that might more effectively eradicate local and systemic micrometastases. Compared with our earlier report of the 5-year survival analysis (4), which showed differences in 5-year DMFS and a trend toward better PFS in the preoperative CRT group, these differences have disappeared at the 10-year mark. The survival curves now exhibit an apparent plateau, indicating the scarce occurrence of disease related events beyond 5 years, which could assist the decision-making in surveillance strategy.

Lymphopenia during cancer treatment has been found to be associated with inferior survival in multiple solid tumors, including non-small cell lung cancer, esophageal cancer, and pancreatic cancer (7). A systematic review and meta-analysis (12) showed that severe radiation-induced lymphopenia was significantly associated with inferior OS in patients receiving radiotherapy for solid tumors, supporting the prognostic relevance of treatment-related lymphocyte depletion. Nevertheless, most previous studies of radiation-induced lymphopenia have focused on absolute lymphocyte counts at specific time points, particularly severe lymphopenia defined by nadir ALC or CTCAE grade. In LA-HNSCC, Price et al. reported that baseline lymphocyte count predicted benefit from concurrent chemotherapy with RT (10), highlighting the potential role of pretreatment immune competence in treatment selection. More recent model-based studies (13, 14) further emphasized that lymphocyte kinetics during and after radiotherapy, including early nadir and post-treatment recovery, are associated with patient outcomes and vary according to tumor site and treatment-related factors. By using on-treatment ALC decline as a dynamic prognostic biomarker, our results are consistent with prior studies, demonstrating that severe lymphopenia during cancer treatment is associated with inferior survival.

Compared with nadir ALC, which reflects the lowest absolute lymphocyte count and may be strongly affected by baseline immune status, ALC decline captures the magnitude of within-patient lymphocyte depletion during radiotherapy. This distinction is clinically relevant because patients with similar nadir ALC values may experience substantially different degrees of immune depletion depending on their pretreatment ALC levels. In our cohort, nadir ALC alone was not significantly associated with OS or PFS, whereas ALC decline remained independently associated with both outcomes after multivariable adjustment. These findings suggest that ALC decline may better reflect individualized treatment-related immune suppression and may be more suitable for dynamic risk stratification during radiotherapy. We also evaluated relative ALC decline whereas did not found its superiority over absolute ALC decline in C-index. Therefore, absolute ALC decline was retained as the primary biomarker considering its simplicity and comparable performance.

The mechanisms underlying RT-induced lymphopenia are multifactorial. Lymphocytes are highly radiosensitive, with an LD_50_ of approximately 2 Gy (7, 9), and can be depleted both directly via irradiation of circulating cells and indirectly through exposure of lymphoid organs and nodal basins. RT may also trigger chronic inflammation, immune exhaustion, and reductions in tumor-infiltrating lymphocytes, all of which may impair anti-tumor immunity (12, 15, 16). Importantly, the degree of lymphocyte depletion is influenced by radiation field size, dose distribution, and whether elective nodal irradiation is employed (17). A recent study demonstrated that conventional dendritic cells (cDC1) within tumor-draining lymph nodes (TDLNs) frequently exhibit high PD-L1 expression, which was associated with CD8^+^ T-cell exhaustion and poor prognosis in oral cancer patients (18). These minimizing incidental radiation to immune-critical structures such as TDLNs may help preserve systemic immune competence. This is particularly relevant in locally advanced HNSCC, where immunotherapy is increasingly incorporated into multimodality treatment and where unnecessary irradiation of lymphatic drainage regions may potentially reducing synergy between RT and immunotherapy. In head and neck cancer, radiotherapy often encompasses extensive cervical nodal basins and lymphatic drainage regions. These areas are immunologically active and may contain TDLNs that are critical for antigen presentation and T-cell priming. Irradiation of these regions may therefore contribute not only to circulating lymphocyte depletion but also to impairment of local antitumor immune activation. Concurrent cisplatin may further exacerbate lymphocyte depletion through systemic cytotoxic effects. In addition, treatment-related lymphopenia may reflect reduced immune cell infiltration or impaired maintenance of effector lymphocytes within the tumor microenvironment, thereby weakening antitumor immune surveillance.

In the immunotherapy era, treatment-related lymphopenia may have even greater clinical relevance. Immune checkpoint inhibitors rely on preserved and functional antitumor lymphocyte populations, and severe depletion of circulating lymphocytes during radiotherapy may compromise systemic immune surveillance and potentially attenuate immunotherapy efficacy. Therefore, lymphocyte preservation should be considered when designing radioimmunotherapy strategies. The optimal sequence of radiotherapy and immunotherapy remains undefined. A recent randomized phase II study comparing concurrent versus sequential pembrolizumab with chemoradiation in locally advanced HNSCC suggested that sequential therapy may be associated with more favorable outcomes than concurrent therapy (19). From a mechanistic perspective, one possible explanation is that concurrent radiotherapy may induce acute lymphocyte depletion during the period when immune checkpoint blockade requires an adequate pool of functional effector lymphocytes. Thus, optimizing the timing, radiation dose, target volume, and lymphocyte-sparing approaches may be critical to maximizing synergy between radiotherapy and immunotherapy. Dynamic ALC decline may also serve as a candidate biomarker for identifying patients at risk of treatment-related immune suppression and for predicting responsiveness to immunotherapy-based combinations. Further prospective studies incorporating serial immune monitoring are warranted to determine whether ALC decline can guide sequencing, dose optimization, and patient selection in radioimmunotherapy trials.

Previous studies have extensively investigated systemic inflammation and nutrition-based indices-such as the SIS, NLR, NAR, and lymphocyte-to-monocyte ratio-as prognostic markers in various solid tumors (16–31). In this study, we comprehensively incorporated a panel of such immune- and nutrition-related indices. Among these, NAR and SIS showed significant associations with outcomes in univariate analysis, whereas they lost significance after multivariable adjustment and ALC decline emerged as an independent prognostic determinant. These findings suggest that although composite indices like NAR and SIS reflect general host status, ALC decline more specifically reflects treatment-related immunosuppression and thus offers greater prognostic robustness in this setting.

To enhance prognostic performance, we integrated ALC decline with other independent predictors into a Cox regression-based nomogram. The model demonstrated good discrimination and calibration, and decision curve analysis indicated meaningful net clinical benefit across a range of risk thresholds. Compared with single-variable predictors, this multivariable approach provides a more individualized estimate of risk, supporting its potential use in tailoring surveillance schedules and selecting patients who may benefit from intensified or alternative treatment regimens. Clinically, patients with marked ALC decline may benefit from closer surveillance and supportive interventions. Potential strategies include reducing unnecessary irradiation of immune-critical structures, minimizing elective nodal irradiation volume, reducing low-dose bath to circulating blood and lymphoid tissues. Additionally, more effort should be made on nutritional status and anti-inflammatory support throughout the course of treatment. For patients identified as high risk by ALC decline and the nomogram, more active follow-up may be considered, particularly during the first five years when most disease-related events occur.

Due to the incomplete data availability, the confounding effect of several critical factors was conducted in the exploratory analyses. Our timing analysis showed recovery status was not significantly associated with survival outcomes and implied the prognostic impact of lymphocyte dynamics was primarily captured by the magnitude of ALC decline during radiotherapy rather than by early post-treatment recovery status. Concerning radiation plan-based parameters, greater ALC decline was not significantly associated with larger GTV, CTV, or PTV volumes, nor with higher target mean dose or prophylactic neck dose. These results suggest that the prognostic association of ALC decline was unlikely to be explained solely by larger tumor burden or larger treatment volume. Concurrent cisplatin may have contributed to treatment-related lymphopenia in the preS-CRT group, as chemotherapy exposure differed between treatment arms by design. However, no significant interaction was observed between treatment group and ALC decline, suggesting that the prognostic association of ALC decline was not clearly restricted to a specific treatment modality. Last, corticosteroid use also had no significant association with survival outcomes after adjustment with ALC decline. Collectively, exploratory analyses supported the independent prognostic value of ALC decline in a supplementary way.

This study has several limitations. First, the optimal cutoff value for ALC decline was derived from ROC analysis within the current cohort and has not been externally validated. Therefore, this threshold should be interpreted as exploratory and dataset-specific. Future prospective multicenter studies are warranted to validate the reproducibility and generalizability of this cutoff. Second, HPV/p16 status was not available for all patients in this retrospective cohort, mainly because HPV or p16 testing was not routinely performed during the earlier treatment period. Based on our recent epidemiological study and the proportion of OPC patients in this cohort, HPV-positive OPC patients were estimated to account for only 10% of the entire cohort (32), precluding reliable HPV-based subgroup analyses. Third, lymphocyte subset data, including CD4+ T cells, CD8+ T cells, regulatory T cells, and natural killer cells, were unavailable. Thus we could not determine which immune cell populations were most strongly associated with survival outcomes. Last, the study cohort predated the routine use of immunotherapy in LA-HNSCC; whether preservation of lymphocyte counts could enhance immunotherapy efficacy warrants investigation. Future studies should validate the prognostic value of ALC decline in multicenter cohorts and in population treated with immunotherapy.

From a clinical perspective, ALC decline is a simple, inexpensive, and non-invasive biomarker that could be incorporated into routine monitoring during RT. Identifying patients with marked ALC decline may allow for timely interventions, such as nutritional optimization, anti-inflammatory strategies, or tailored radiation planning to reduce unnecessary lymphoid irradiation. Furthermore, in the context of emerging immunotherapy combinations, minimizing treatment-related lymphopenia through precision RT design may help preserve immune function and potentially improve therapeutic synergy. Future studies should validate the prognostic value of ALC decline in multicenter cohorts and in population treated with immunotherapy. Prospective studies investigating whether interventions designed to prevent or mitigate lymphopenia can improve outcomes are also highly warranted. Such interventions may include lymphocyte-sparing radiotherapy, optimization of elective nodal irradiation, nutritional and immune-supportive strategies, and integration with immunotherapy. Comprehensive prognostic models incorporating lymphocyte subsets, tumor immune infiltration, circulating immune markers, and immune checkpoint expression may further improve individualized risk prediction.

## Conclusion

In summary, this *post-hoc* analysis of a randomized phase III trial demonstrates promising 10-year survival outcomes in LA-HNSCC after receiving preoperative RT or CRT followed by radical surgery or boosted RT. More remarkably, treatment-related decline in ALC during radiotherapy is an independent prognostic factor for both progression-free and overall survival in patients with LA-HNSCC, underscoring its potential as a practical biomarker for risk stratification. Prospective validation and interventional studies are warranted to determine whether lymphocyte-sparing strategies and supportive measures can translate into survival benefits in head and neck cancers.

## Data Availability

The original contributions presented in the study are included in the article/**Supplementary Material**. Further inquiries can be directed to the corresponding authors.

## Supplementary material

Supplementary Figure 1 Restricted cubic spline analyses of immune-nutritional indices and overall survival. Restricted cubic spline curves showing the associations of **(A)** ALC decline, **(B)** neutrophil-to-albumin ratio (NAR), and **(C)** systemic inflammation score (SIS) with overall survival.

Supplementary Figure 2 Interaction analyses between treatment group and ALC decline. Interaction analyses evaluating the association between continuous absolute lymphocyte count (ALC) decline and survival outcomes according to treatment group. ALC decline was modeled as a continuous variable. **(A)** Overall survival. **(B)** Progression-free survival.

Supplementary Figure 3 Subgroup analyses of the prognostic effect of high ALC decline. Forest plots showing the association between high ALC decline and survival outcomes across clinically relevant subgroups. **(A)** Progression-free survival subgroup analysis. **(B)** Overall survival subgroup analysis.

Supplementary Figure 4 Time-dependent predictive performance of the prognostic model. Time-dependent AUC for progression-free survival.

Supplementary Figure 5 Time-dependent ROC curves comparing absolute and relative ALC decline models. Time-dependent receiver operating characteristic (ROC) curves comparing the predictive performance of models based on absolute ALC decline and relative ALC decline. The upper row shows ROC curves for overall survival (OS), and the lower row shows ROC curves for progression-free survival (PFS). Columns represent 1-year, 3-year, and 5-year prediction time points.

Supplementary Figure 6 Individual longitudinal ALC trajectories during and after radiotherapy. Individual longitudinal absolute lymphocyte count (ALC) trajectories from the start of radiotherapy through post-treatment follow-up.
