# Supplementary material for: Treatment-related lymphopenia predicts long-term survival in locally advanced head and neck cancer: Post-Hoc analysis of a randomized phase III trial
Source: Front Immunol. 2026 Aug 31;17:1805437. doi: 10.3389/fimmu.2026.1805437 (PMC13572264; doi:10.3389/fimmu.2026.1805437)

Supplementary figure 1

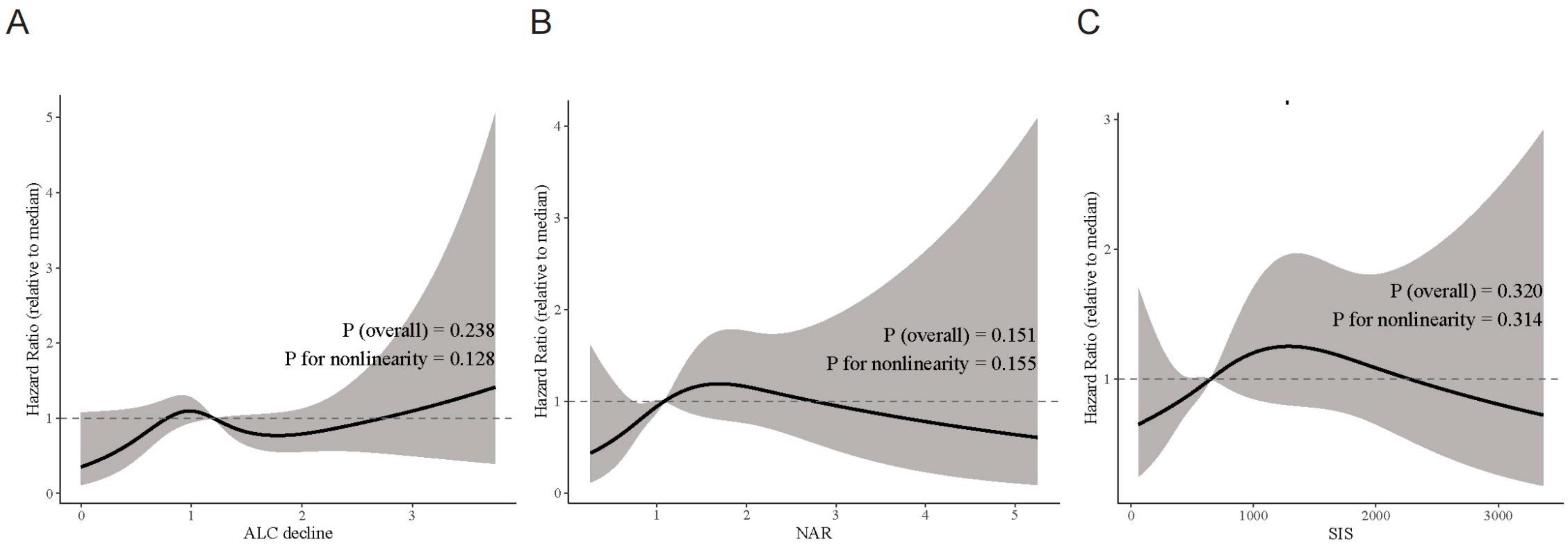

Supplementary Figure 2

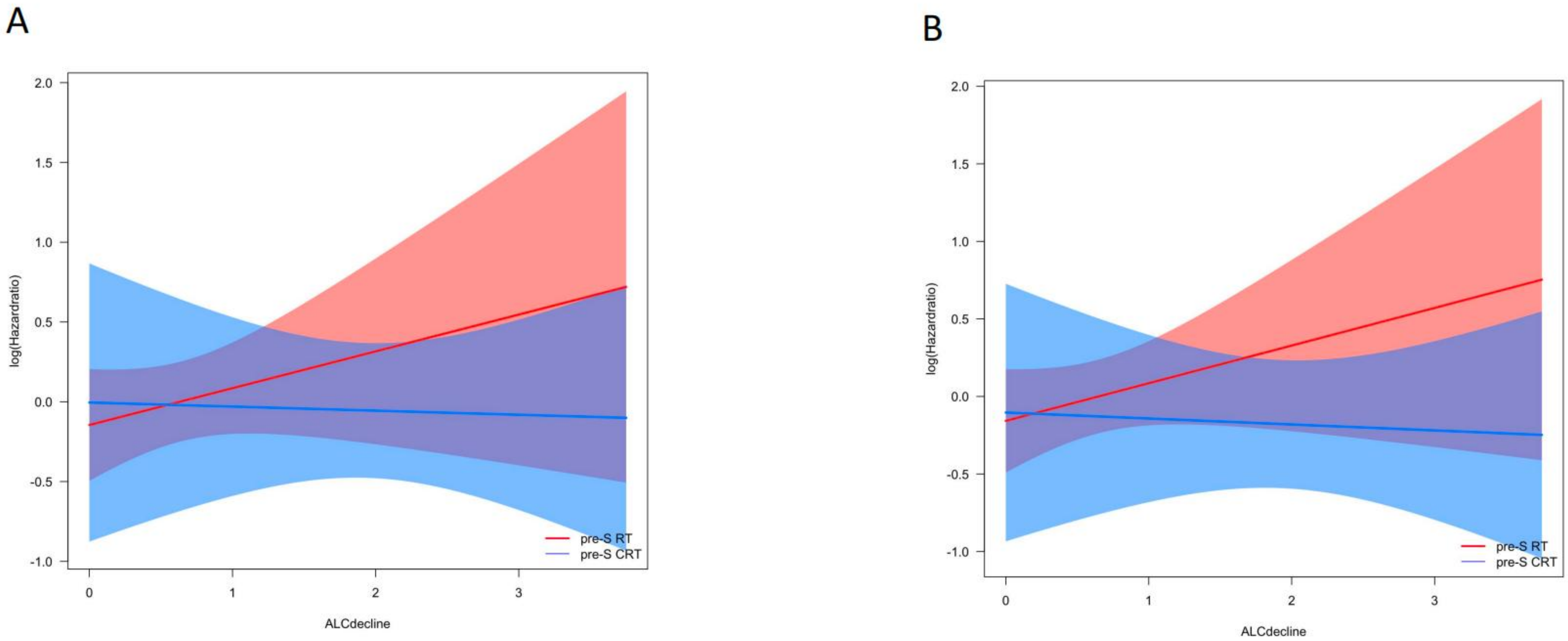

Supplementary figure 3

A

Subgroup	Events/Total (Low ALC decline)	Events/Total (High ALC decline)	spacer	HR (95% CI)	P value	P for interaction
	No. of patients with events/total no.					
PFS subgroup						
Treatment group						0.918
preS-RT	11/31	53/87		1.86 (0.97-3.57)	0.061	
preS-CRT	5/19	39/85		1.96 (0.77-4.97)	0.157	

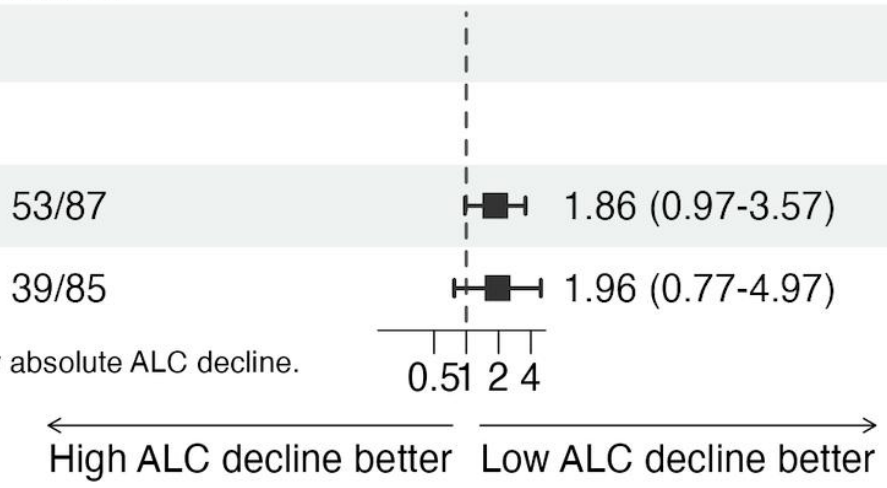

Subgroup analysis for PFS; HR compares high vs low absolute ALC decline.

B

Subgroup	Events/Total (Low ALC decline)	Events/Total (High ALC decline)	spacer	HR (95% CI)	P value	P for interaction
	No. of patients with events/total no.					
OS subgroup						
Treatment group						0.589
preS-RT	13/31	57/87		1.65 (0.90-3.01)	0.105	
preS-CRT	9/19	50/85		1.29 (0.63-2.62)	0.489	

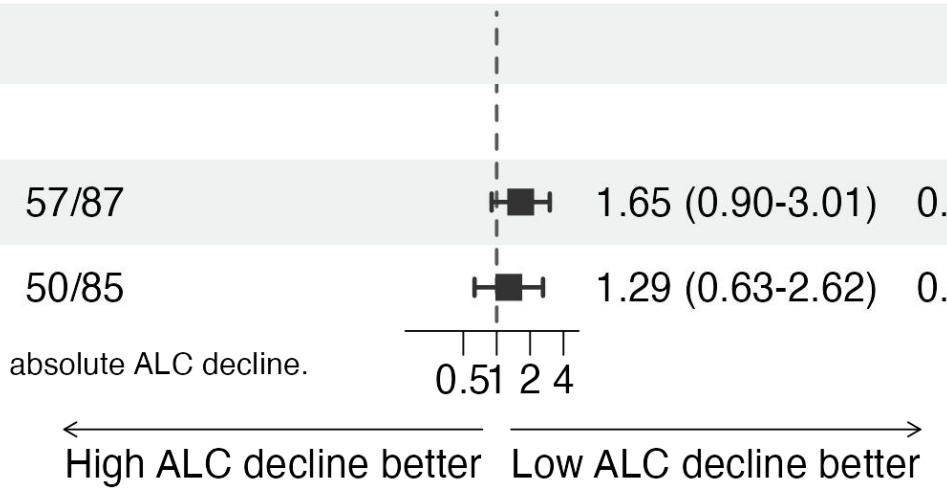

Subgroup analysis for OS; HR compares high vs low absolute ALC decline.

Supplementary figure 4

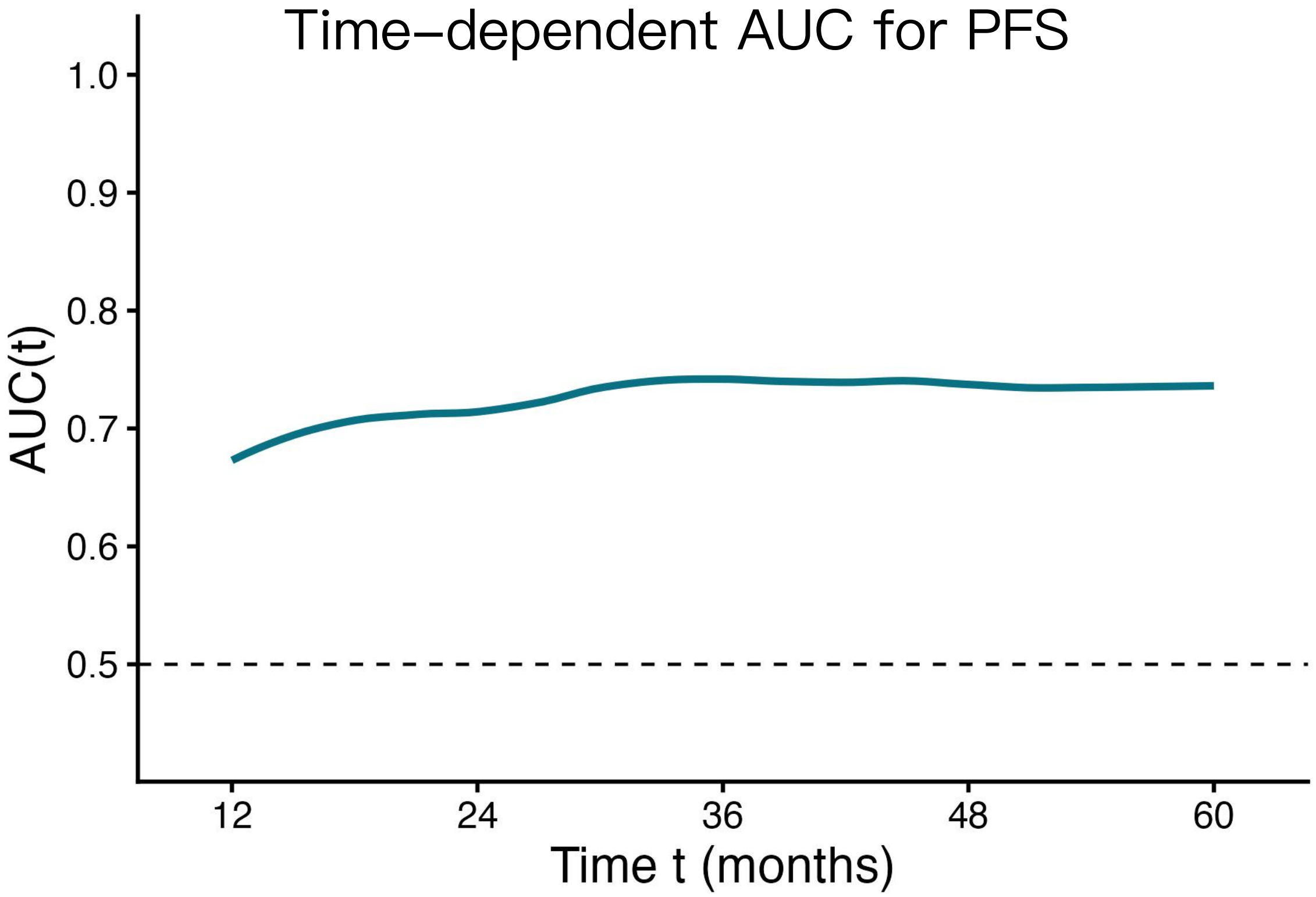

Supplementary figure 5

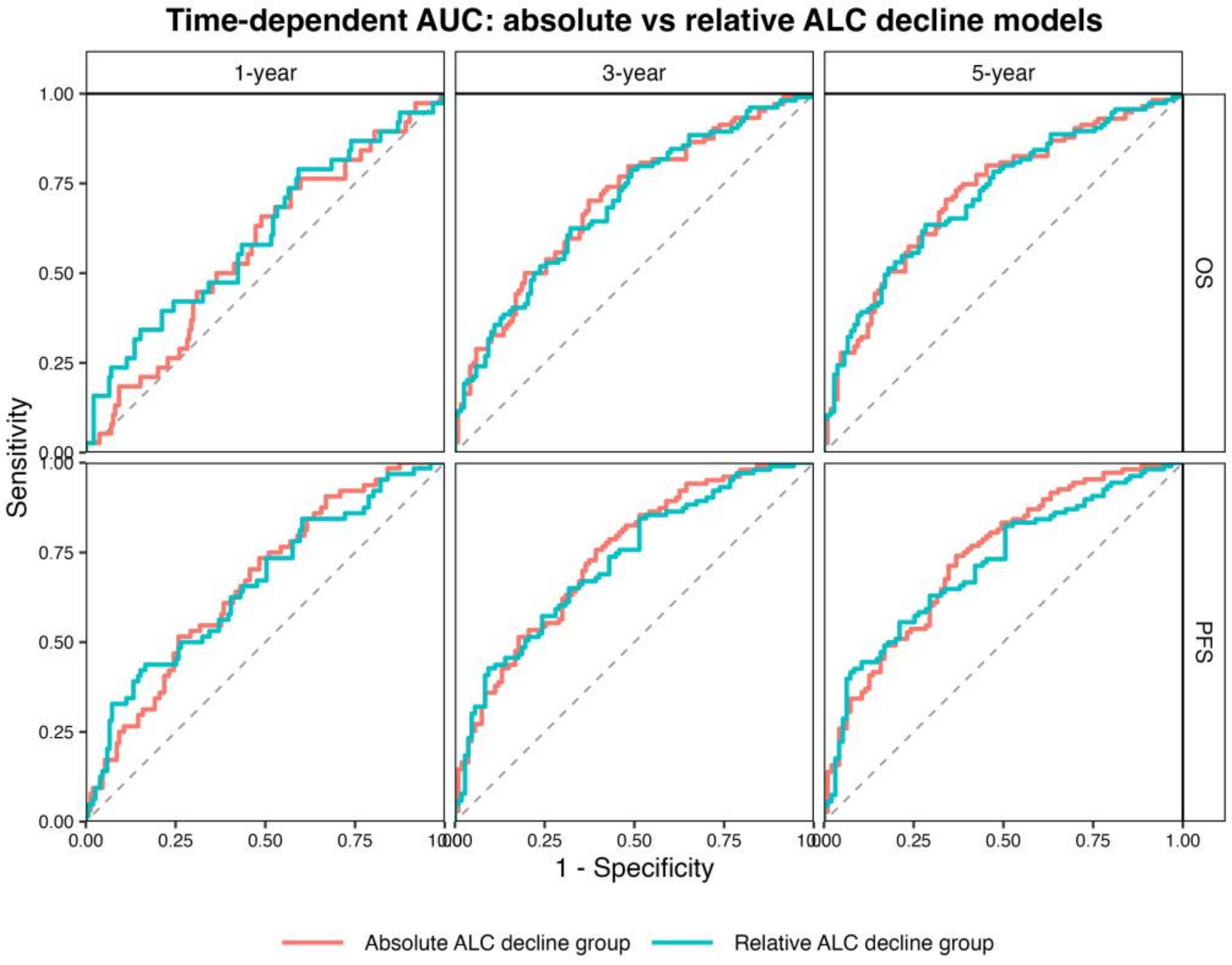

Supplementary figure 6

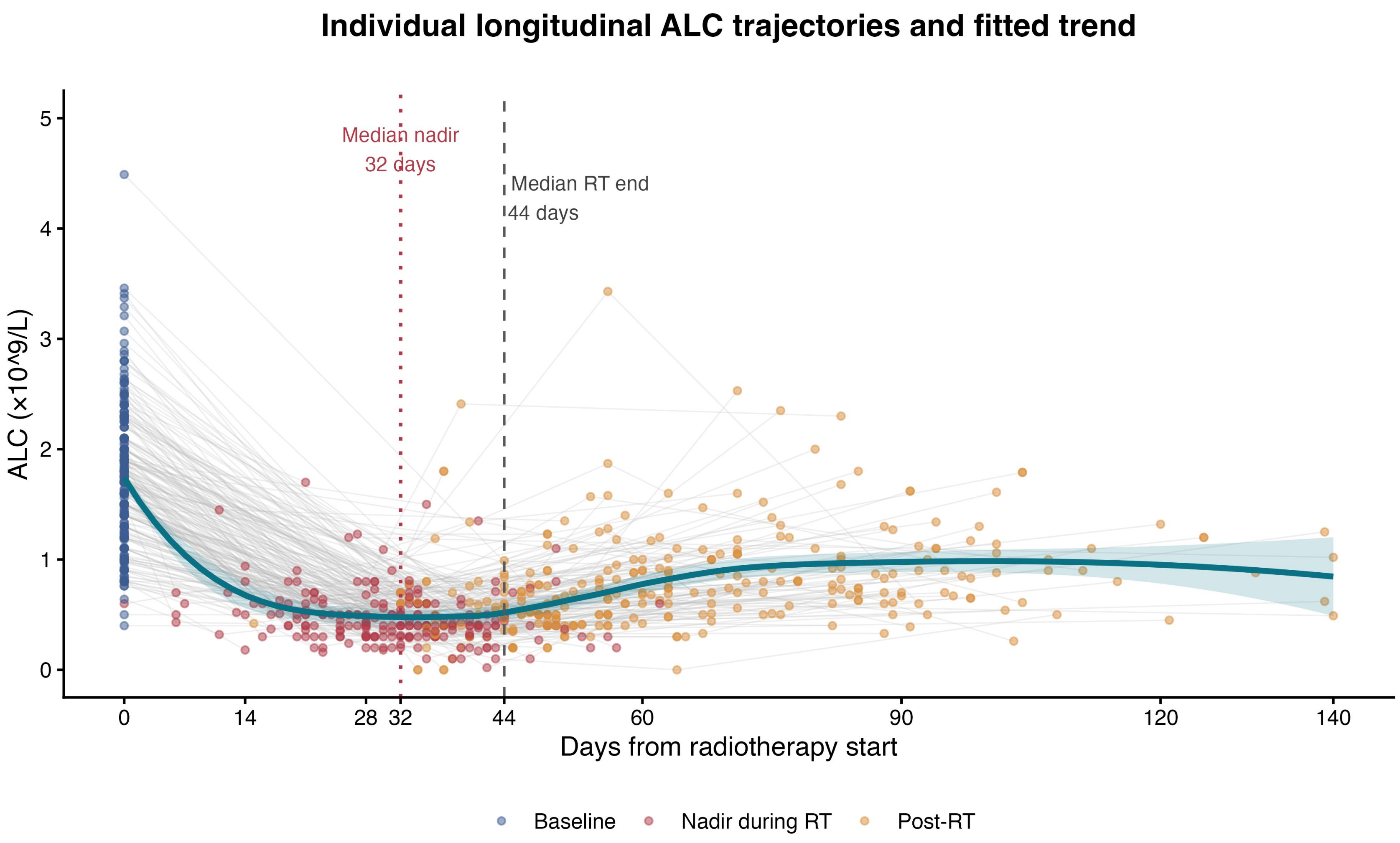

Supplement: Supplementary file 1 [file Image1.pdf]
