# Supplementary material for: Treatment-related lymphopenia predicts long-term survival in locally advanced head and neck cancer: Post-Hoc analysis of a randomized phase III trial
Source: Front Immunol. 2026 Aug 31;17:1805437. doi: 10.3389/fimmu.2026.1805437 (PMC13572264; doi:10.3389/fimmu.2026.1805437)
Supplement: Supplementary file 2 [file Table1.docx]

| **Supplementary table 1. Definition and formula of immune and nutritional indices.** | |
| --- | --- |
| Index | Definition / Formula |
| ALC decline | Pretreatment ALC − nadir ALC during radiotherapy (RT) |
| NLR  (Neutrophil-to-Lymphocyte Ratio) | Absolute neutrophil count / absolute lymphocyte count |
| PLR  (Platelet-to-Lymphocyte Ratio) | Platelet count / absolute lymphocyte count |
| PNI  (Prognostic Nutritional Index) | Albumin (g/L) + 5 × lymphocyte count (×10⁹/L) |
| NAR  (Neutrophil-to-Albumin Ratio) | Absolute neutrophil count / albumin (g/L) |
| SIS  (Systemic Inflammation Score) | Platelet count × (neutrophil count / lymphocyte count) |
| GNRI  (Geriatric Nutritional Risk Index) | 1.489 × albumin (g/L) + 41.7 × (body weight / ideal body weight) |
| BMI  (Body Mass Index) | Body weight (kg) / height² (m²) |
| AHR  (Albumin-to-Hemoglobin Ratio) | Albumin (g/dL) / hemoglobin (g/dL) |
| SAR  (Serum Iron-to-Albumin Ratio) | Serum iron (µg/dL) / albumin (g/dL) |
| PAR  (Platelet-to-Albumin Ratio) | Platelet count (/µL) / albumin (g/dL) |
| WAPNI  (Weight-Adjusted PNI) | (10 × albumin (g/dL) + 0.005 × lymphocyte count (/mm³)) / BMI |
| CIMI  (Combined Iron Metabolism Index) | (Serum iron + transferrin) / albumin |
| NISI  (Nutritional-Inflammatory Status Index) | (Albumin + hemoglobin) / (platelet count + transferrin) |
| Nadir ALC | Lowest lymphocyte count during RT |

| **Supplementary Table 2. Global Schoenfeld residual tests for proportional hazards assumption.** | | | |
| --- | --- | --- | --- |
| Outcome | Model | Global Schoenfeld p value | Interpretation |
| OS | Absolute ALC decline model | 0.400 | No significant global PH violation |
| PFS | Absolute ALC decline model | 0.646 | No significant global PH violation |

| **Supplementary table 3. Interaction analyses between treatment group and continuous ALC decline using restricted cubic spline Cox models.** | | | | |
| --- | --- | --- | --- | --- |
| Outcome | Effect tested | Chi-Square | df | P value |
| PFS | Txgroup (main effect) | 4.12 | 4 | 0.3900 |
| PFS | ALCdecline × Txgroup (interaction) | 1.24 | 3 | 0.7444 |
| OS | Txgroup (main effect) | 3.41 | 4 | 0.4910 |
| OS | ALCdecline × Txgroup (interaction) | 2.28 | 3 | 0.5172 |
| Abbreviations: PFS, progression-free survival; OS, overall survival; df, degrees of freedom. Note: Results were derived from multivariable Cox models in which ALC decline was modeled as a continuous variable using restricted cubic splines. Models were adjusted for T stage and N stage. The interaction test evaluates whether the association between continuous ALC decline and survival outcomes differed by treatment group. | | | | |

| **Supplementary table 4 Interaction analyses between treatment group and categorized ALC decline for OS and PFS** | | | | | | |
| --- | --- | --- | --- | --- | --- | --- |
| **Outcome** | **Characteristic** | **N** | **Event N** | **HR** | **95% CI** | **p-value** |
| **OS** | **Treatment group** |  |  |  |  |  |
|  | preS-RT | 118 | 70 | Reference | Reference |  |
|  | preS-CRT | 104 | 59 | 1.19 | 0.49-2.89 | 0.699 |
|  | **ALC decline group** |  |  |  |  |  |
|  | Low ALC decline | 50 | 22 | Reference | Reference |  |
|  | High ALC decline | 172 | 107 | 1.95 | 1.05-3.62 | 0.035 |
|  | **Treatment × ALC decline** |  |  |  |  |  |
|  | preS-CRT × High ALC decline | 85 | 50 | 0.67 | 0.25-1.75 | 0.410 |
| **PFS** | **Treatment group** |  |  |  |  |  |
|  | preS-RT | 118 | 76 | Reference | Reference |  |
|  | preS-CRT | 104 | 62 | 0.97 | 0.40-2.31 | 0.940 |
|  | **ALC decline group** |  |  |  |  |  |
|  | Low ALC decline | 50 | 23 | Reference | Reference |  |
|  | High ALC decline | 172 | 115 | 2.07 | 1.13-3.78 | 0.018 |
|  | **Treatment × ALC decline** |  |  |  |  |  |
|  | preS-CRT × High ALC decline | 85 | 53 | 0.74 | 0.29-1.91 | 0.535 |

| **supplementary table 5 Predictive performance of absolute and relative ALC decline models for OS and PFS.** | | | | | |
| --- | --- | --- | --- | --- | --- |
| Outcome | Model | C-index | 1-year AUC | 3-year AUC | 5-year AUC |
| OS | Absolute ALC decline group | 0.632 | 0.571 | 0.700 | 0.721 |
| OS | Relative ALC decline group | 0.636 | 0.606 | 0.693 | 0.717 |
| PFS | Absolute ALC decline group | 0.660 | 0.676 | 0.739 | 0.738 |
| PFS | Relative ALC decline group | 0.654 | 0.660 | 0.726 | 0.716 |

Relative ALC decline was defined as (baseline ALC − nadir ALC) / baseline ALC. The optimal cutoff for relative ALC decline was **0.887**, corresponding to an **88.7% decline**, based on ROC analysis using 3-year OS as the endpoint. ALC, absolute lymphocyte count; OS, overall survival; PFS, progression-free survival; AUC, area under the curve.

| **Supplementary table 6 Timing of nadir ALC during radiotherapy.** | |
| --- | --- |
| Parameter | Value |
| Patients analyzed | 222 |
| Time from RT start to nadir ALC, days | 32, IQR 25–38 |
| Time from nadir ALC to RT end, days | 10, IQR 4–18 |
| Relative timing within RT course | 76%, IQR 58%–88% |
| Nadir ALC within final 2 weeks of RT | 139/222, 62.6% |
| Nadir ALC within final 1 week of RT | 87/222, 39.2% |
| Relative timing within the RT course was calculated as the time from RT start to nadir ALC divided by the total RT duration. ALC, absolute lymphocyte count; RT, radiotherapy; IQR, interquartile range. | |

| **Supplementary table 7 Association between one-month lymphocyte recovery status and survival outcomes.** | | | | |
| --- | --- | --- | --- | --- |
| Outcome | recovered n/events | persistent n/events | HR，persistent vs recovered | P value |
| OS | 87 / 44 | 85 / 51 | 1.23，95% CI 0.82-1.84 | 0.321 |
| PFS | 87 / 40 | 85 / 43 | 1.17，95% CI 0.76-1.80 | 0.477 |
| LRFS | 87 / 28 | 85 / 30 | 1.16, 95% CI 0.69-1.93 | 0.583 |
| DMFS | 87 / 44 | 85 / 51 | 1.24, 95% CI 0.83-1.85 | 0.301 |
| Lymphocyte recovery was assessed approximately one month after completion of radiotherapy. Recovered was defined as one-month post-treatment ALC ≥0.8 × 10⁹/L, whereas persistent lymphopenia was defined as one-month post-treatment ALC <0.8 × 10⁹/L. ALC, absolute lymphocyte count; HR, hazard ratio; CI, confidence interval; OS, overall survival; PFS, progression-free survival; LRFS, locoregional relapse-free survival; DMFS, distant metastasis-free survival. | | | | |

| **Supplementary table 8 Comparison of target volumes and dosimetric parameters according to ALC decline group.** | | | |
| --- | --- | --- | --- |
| Parameter | Low ALC decline ≤0.74 ×10⁹/L, median (IQR) | High ALC decline >0.74 ×10⁹/L, median (IQR) | P value |
| GTVp volume, cm³ | 28.68 (14.80-41.45) | 25.22 (12.64-49.70) | 0.865 |
| GTVp Dmean, cGy | 7000.00 (6196.20-7300.40) | 6998.00 (5750.50-7228.13) | 0.330 |
| GTVnd volume, cm³ | 10.05 (3.25-22.20) | 9.75 (5.68-26.28) | 0.445 |
| GTVnd Dmean, cGy | 6996.00 (5896.80-7159.20) | 7000.00 (5704.14-7140.35) | 0.911 |
| CTV1 volume, cm³ | 430.80 (360.73-543.17) | 435.80 (373.31-535.50) | 0.997 |
| CTV1 Dmean, cGy | 6000.00 (5681.60-6555.80) | 6000.00 (5000.00-6270.40) | 0.377 |
| CTV2 volume, cm³ | 52.70 (37.20-73.80) | 53.78 (32.61-87.78) | 0.883 |
| CTV2 Dmean, cGy | 5138.00 (5096.00-5385.80) | 5100.00 (5000.00-5422.40) | 0.642 |
| PTV1 volume, cm³ | 615.58 (499.86-736.05) | 638.11 (531.58-753.60) | 0.733 |
| PTV1 Dmean, cGy | 6000.00 (5592.50-6468.30) | 6000.00 (5000.00-6347.00) | 0.421 |
| PTV2 volume, cm³ | 83.70 (60.73-112.09) | 90.68 (49.24-132.52) | 0.874 |
| PTV2 Dmean, cGy | 5138.00 (5096.00-5327.30) | 5100.00 (5000.00-5361.65) | 0.606 |
| Values are presented as median (IQR). P values were calculated using the Mann–Whitney U test. ALC decline was defined as pretreatment ALC minus nadir ALC during radiotherapy. ALC, absolute lymphocyte count; GTVp, primary gross tumor volume; GTVnd, nodal gross tumor volume; CTV, clinical target volume; PTV, planning target volume; Dmean, mean dose; IQR, interquartile range. | | | |

| **Supplementary table 9 Cox models including high ALC decline and corticosteroid use for survival outcomes.** | | | | |
| --- | --- | --- | --- | --- |
| Outcome | High ALC decline, HR (95% CI) | P value | Corticosteroid use, HR (95% CI) | P value |
| OS | 1.54 (0.97-2.45) | 0.067 | 0.70 (0.43-1.15) | 0.157 |
| PFS | 1.67 (1.07-2.63) | 0.025 | 0.72 (0.45-1.15) | 0.171 |
| LRFS | 1.69 (0.93-3.07) | 0.085 | 0.74 (0.40-1.36) | 0.329 |
| DMFS | 1.56 (0.98-2.47) | 0.061 | 0.70 (0.43-1.15) | 0.162 |
| Cox models included high ALC decline and corticosteroid use to explore whether recorded corticosteroid exposure attenuated the association between ALC decline and survival outcomes. High ALC decline was defined as ALC decline >0.74 ×10⁹/L. Corticosteroid use was analyzed as yes versus no. | | | | |
